# Supplementary material for: Predictive value of ultrasound doppler parameters in neoadjuvant chemotherapy response of breast cancer: Prospective comparison with magnetic resonance and mammography
Source: PLoS One. 2024 Jun 4;19(6):e0302527. doi: 10.1371/journal.pone.0302527 (PMC11149875; doi:10.1371/journal.pone.0302527)
Supplement: Attachment — Submitted filename: recommendation.docx [file pone.0302527.s002.docx]

The study is well written, well structured and logical. I have a few questions about the part of methods:

1. Although the method of ultrasonic examination is explained in this paper, it is not detailed enough. As we all know, the repeatability of ultrasound examination is not good. If the final values of multiple ultrasound examination parameters mentioned in this paper are clear, such as ED, PS, MS, PI, RI, and lesion elasticity value, the results can be obtained. How to solve the problem of inspection repeatability in research?
2. In the pathological classification of RCB, only descriptive words are used to distinguish RCB-I, RCB-ii, and RCB-III. Is there any specific quantitative index to replace descriptive words as the classification standard?
