# Supplementary material for: Predictive value of ultrasound doppler parameters in neoadjuvant chemotherapy response of breast cancer: Prospective comparison with magnetic resonance and mammography
Source: PLoS One. 2024 Jun 4;19(6):e0302527. doi: 10.1371/journal.pone.0302527 (PMC11149875; doi:10.1371/journal.pone.0302527)
Supplement: Attachment — Submitted filename: Rebuttal_Letter_Plos_Livia_07032024.docx [file pone.0302527.s003.docx]

Dr . Daniele Ugo Tari. M.D.

*Academic Editor*

**PLOS ONE**

**Subject: Submission of Manuscript – "Predictive Value of Ultrasound Doppler Parameters in Neoadjuvant Chemotherapy Response of Breast Cancer: prospective comparison with Magnetic Resonance and Mammography"**

Conz, L; Jales, R.M.; Doria, M.T.; Melloni, I.; Lyrio, C.A.C.; Menossi, C.; Derchain, S.F.M.; Sarian, L.O.

Dear Dr. Tari,

I am pleased to submit the revised version of four manuscript entitled "Predictive Value of Ultrasound Doppler Parameters in Neoadjuvant Chemotherapy Response of Breast Cancer: prospective comparison with Magnetic Resonance and Mammography" for further consideration for publication in PLOS ONE.

In this revised version, we have addressed each of the three reviewers’ concerns. In the following pages, you will find a detailed rebuttal of such concerns and a description of the modifications made to the original manuscript.

We hope that the adjustments made enhance the manuscript’s suitability for publication in PLOS ONE. Regardless of the final decision, we are grateful to the time and effort invested in reviewing our paper.

We look forward to your feedback.

Sincerely,

Luis Otavio Sarian

M.D., PhD, Full Professor - Department of Obstetrics and Gynecology, Division of Breast and Gynecologic Oncology

State University of Campinas (Unicamp), Campinas, São Paulo, Brazil

**Response to reviewers:**

**Reviewer #1**

**Comments:** *“The manuscript is interesting and well designed. Introduction is good written.
Materials and methods are good designed. Results are good illustrated
Discussion is good written and include the conclusion of the study”*

**Response:** Thank you very much for the time and effort in reviewing our manuscript. We appreciate your positive feedback and encouraging words.

**Reviewer #2:**

**Comment 1**: *“The study is well written, well structured and logical. I have a few questions about the part of methods: Although the method of ultrasonic examination is explained in this paper, it is not detailed enough. As we all know, the repeatability of ultrasound examination is not good. If the final values of multiple ultrasound examination parameters mentioned in this paper are clear, such as ED, PS, MS, PI, RI, and lesion elasticity value, the results can be obtained. How to solve the problem of inspection repeatability in research?”*

**Response to comment 1:** Thank you very much for reviewing our manuscript. We appreciate your positive comments as well as your encouraging words. Indeed, the question of whether the parameters used in our proposed protocol are repeatable and reproducible is fair and thoughtful. Fortunately, the reproducibility of the measurements of ED, OS, MS, PI, and RI is of least concern to us, as they are fully automated. The challenge lies in identifying the masses for which such parameters will be measured; however, the requisite ability can be acquired after a few months of training in a specialized imaging center and is far from requiring expert-level proficiency. Thus, we are confident that the measurements performed for our study can be easily reproduced in other centers, provided a reasonable amount of professional training is obtained, and capable equipment is available. These aspects have now been clarified and discussed in the “Discussion” section of the revised manuscript.

**Comment 2:** *“In the pathological classification of RCB, only descriptive words are used to distinguish RCB-I, RCB-ii, and RCB-III. Is there any specific quantitative index to replace descriptive words as the classification standard?”.*

**Response to comment 2:** Another thoughtful comment. We have now referenced the MD Anderson reference guide used to render the RCB diagnoses.

**Reviewer #3:**

**Comment 1**: *“The article is innovative. Simple and non-invasive interventions were used in the neoadjuvant process: ultrasound Doppler and shear wave imaging were used to evaluate the neoadjuvant treatment of intracavitary or non-intracavitary tumors, respectively, to observe the tumor prognosis.”*

**Response to comment 1:** Thank you for your positive comments. We appreciate your acknowledgment of the innovative aspects of our study.

**Comment 2:** *“The sum of the number of cases in line 13 of the method paragraph in the article is 172 cases, not 173 cases. Please give an explanation”*

**Response to comment 2:** We apologize for this error, and the surgery counts have now been corrected to 68 mastectomies and 105 conservative surgeries, totaling 173 patients.

**Comment 3:** *“Are the Doppler indicators averaged in the value of non-intracavitary tumors?”*

**Response to comment 3**: No. The Doppler indicators were automatically calculated using the absolute Doppler velocimetry obtained by insonating the central portion of the breast lesions, as perceived by the sonographer. The Doppler variables thus refer to the largest vessels identified in each tumor, as first identified using color Doppler and then measured using spectral Doppler. The only correction applied was for the angle of insonation, for values <60 degrees. These clarifications were added to the Methods section, Ultrasound.

**Comment 4:** *“Is the area of interest measured by shear wave completely covering the lump or taking small pieces within the lump?”*

**Response to comment 4**: We took two measurements for each lump, one in the central region of the tumor, and another on parenchyma surrounding the tumor. These details have been added to the manuscript.
